# Supplementary figures and images for: The Use of Sand Substrate Modulates Dominance Behaviour and Brain Gene Expression in a Flatfish Species
Source: Animals (Basel). 2023 Mar 8;13(6):978. doi: 10.3390/ani13060978 (PMC10044175; doi:10.3390/ani13060978)

Supplementary Figure S1

A

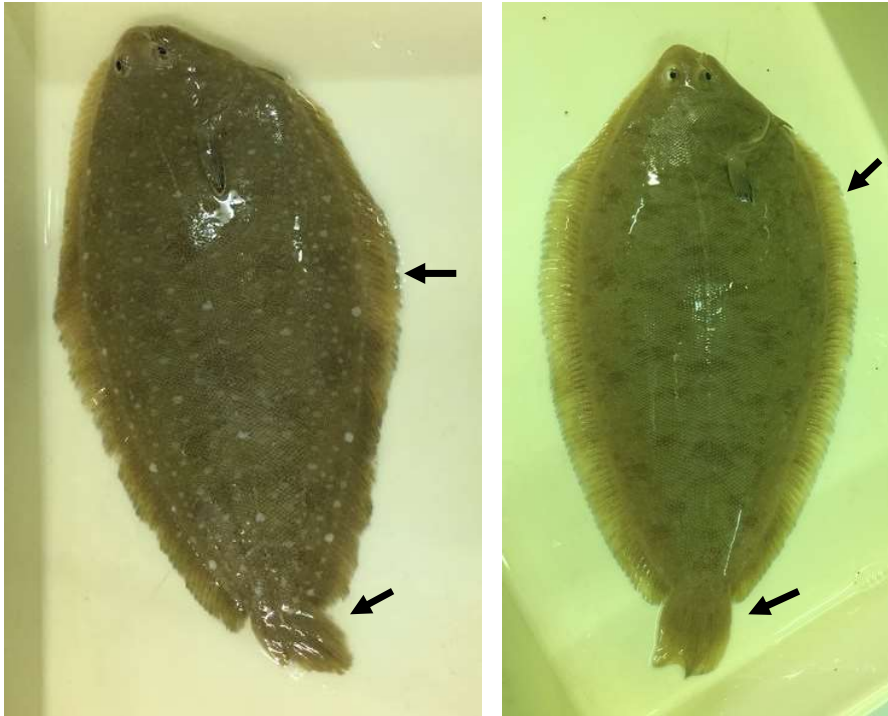

B

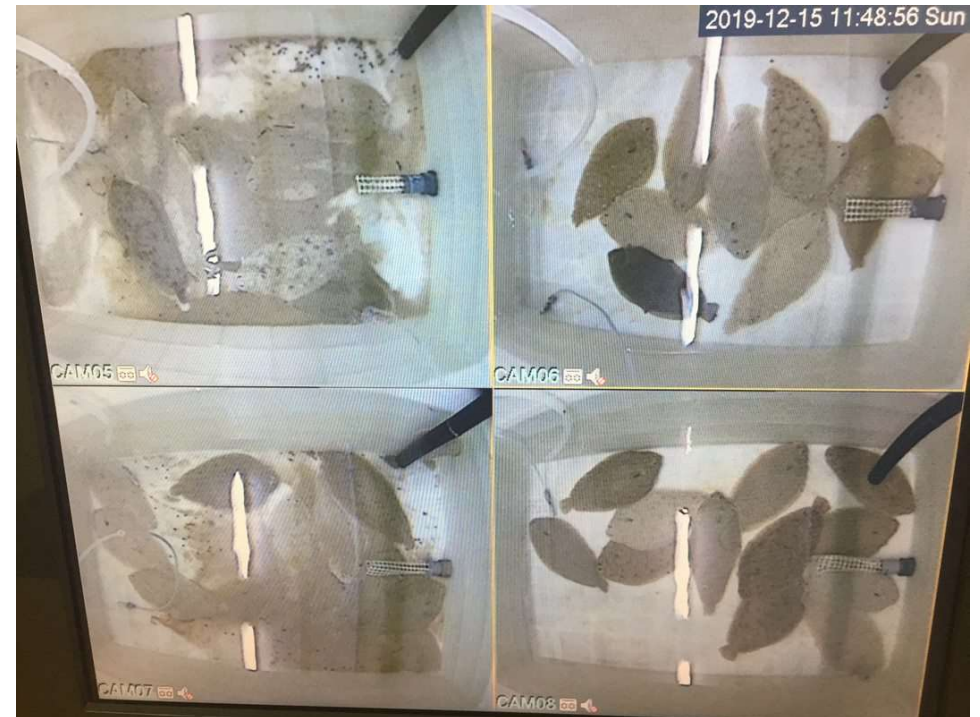

Supplement: Supplementary file 1 [file animals-13-00978-s001.zip › animals-2242180-supplementary.pdf]
